# Multimodality Imaging Training Curriculum — Parts II and III

**DOI:** 10.1007/s13244-012-0172-1

**Published:** 2012-05-09

**Authors:** European Association of Nuclear Medicine (EANM), European Society of Radiology (ESR)

**Affiliations:** 1 Hollandstrasse 14/Mezzanine, A-1020 Vienna, Austria; 2 Neutorgasse 9/2, A-1010 Vienna, Austria

**Introduction**

This publication is a continuation of the document entitled "Multimodality Imaging Training Curriculum — General Recommendations" which was published in the ESR journal *Insights into Imaging* (Insights Imaging 2(2): 99–101, DOI: 10.1007/s13244-011-0067-6) and in EJNMMI (DOI 10.1007/s00259-011-1804-2).

The following sections of the document (Part II and III) list specific knowledge requirements for appropriate training in multimodality imaging for those whose background is in nuclear medicine and radiology respectively (PET/CT, SPECT/CT).

Apart from specified training items it is understood that practising multimodality imaging requires certain skills above and beyond those enumerated under specific pathology areas. These include knowledge of emergency imaging findings as relevant to each specific organ system (BK = Basic Knowledge), interpretation and management of unexpected findings in the imaging of each organ system (CS = Core Skills), an understanding of the process of justification of requests for imaging investigation and appropriateness criteria (CS), creation of a structured, coherent, and clinically relevant imaging report (CS), an understanding of the medico-legal implication of imaging practice (CS), basics of oncogenesis including elements of cell biology and genetics (BK), principles of systemic, regional and targeted therapies (BK), principles and application of screening methodologies (BK).

**Members of the combined EANM/ESR Curriculum Working Group:**

Chairpersons: W.H. Knapp (EANM), É. Breatnach (ESR)

**EANM Members:**

C. Hoefnagel, D. Huić, T. Nunan, F. Verzijlbergen

**ESR Members:**

K. Åhlström Riklund, G. P. Krestin, M. F. Reiser

**PART II**

**Nuclear Medicine Curriculum for those whose Training Background is in Radiology**

**Preamble to Part II**

In 2011 the “Multimodality Imaging Training Curriculum – General Recommendations” were published in the *EJNMMI* (Eur J Nucl Med Mol Imaging DOI 10.1007/s00259-011-1804-2) and in *Insights into Imaging* (Insights Imaging 2(2): 99-101, DOI: 10.1007/s13244-011-0067-6). Both the European Association of Nuclear Medicine and the European Society of Radiology committed themselves to writing more detailed recommendations regarding skills and knowledge requirements for nuclear medicine physicians in radiology and radiologists in nuclear medicine. Parts II & III of the Multimodality Imaging Training Curriculum were endorsed by the ESR Executive Council in June 2011, as well as the EANM Executive Committee, the EANM Advisory Council and on February 26, 2012 by the extraordinary EANM Delegates Assembly.

The Multimodality Imaging Training Curriculum is a joint European initiative between the EANM and the ESR to define the scope of training for medical specialists in hybrid imaging. The curriculum is a framework outline that may be used by member societies where appropriate to form the basis for discussion on the optimisation of further training of medical specialists.

This curriculum cannot be used to legally justify changes to the organisation, overall specialist training or reimbursement regulations in any member state.

**Nuclear Medicine Curriculum for those whose Training Background is in Radiology**

The following outlines a check list of nuclear medicine training requirements for radiologists to ensure the appropriate level of knowledge is obtained for performing and reading hybrid imaging such as PET/CT and SPECT/CT within a 2-year training period. This checklist differentiates three levels of competence: basic knowledge (BK), detailed knowledge (DK) and core skills (significant practical experience required, CS).

**A. Required general training in nuclear medicine**

***Production and properties of radionuclides*** **(BK)**

The production of radionuclides by cyclotrons, radionuclide generators and reactors

The production of PET nuclides by cyclotrons and radionuclide generators

Practical issues related to the use of short-lived radionuclides

Practical issues related to the use of generators to produce radionuclides

The physical properties of commonly used diagnostic radionuclides (physical half-life, photon energy)

***Tracer principles and techniques*** **(BK)**

The basic principles of tracer studies

The kinetics of radioactive tracers used in nuclear medicine

The use of principles of kinetics and modelling techniques to calculate parameters such as glomerular filtration rate

Errors associated with quantitative measurements

***Radiopharmacy***

The principles of localisation of radiopharmaceuticals **(BK)**

Different formulations used in nuclear medicine **(BK)**

Cell labelling techniques **(BK)**

The physicochemical and biological properties of different radiopharmaceuticals in routine clinical practice (biological half-life, biokinetics, dosimetry) **(DK)**

***Legislation***

Regulations relating to radiation protection **(DK)**

Specific regulations relating to the use of radionuclides **(DK)**

Specific regulations relating to the manufacture of radionuclides **(BK)**

National and international regulatory requirements for the practice of nuclear medicine **(BK)**

Radiopharmaceutical transport **(BK)**

Waste disposal **(BK)**

***Instrumentation***

Nuclear medicine equipment **(BK)**

Principles of gamma camera and PET systems **(DK)**

Nuclear physics **(BK)**

***Radiation protection and other safety issues*** **(DK)**

**B. Specific studies by organ**

Understanding of the role of nuclear medicine procedures in the clinical management of the relevant disease entities and the various protocols used **(DK)**

Understanding of the complementary imaging studies and the appropriate use of the various procedures in the management of patients **(DK)**

Normal anatomy, physiology and pathophysiology **(BK)**

***Musculoskeletal*** **(DK)**

Benign bone disease (fractures, osteomyelitis, Paget's disease, fibrous dysplasia, sport injuries)

Malignant bone disease (primary and secondary)

Joint disease (arthritis, infected joints, infected prostheses)

Bone marrow studies

Understanding of the role of SPECT/CT in addition to planar scintigrams and of PET/CT using FDG, F-18- fluoride and other relevant agents commonly used in musculoskeletal diseases

Patient preparation and procedural aspects depending on the clinical question, e.g. three-phase imaging

***Pulmonary***

Ventilation/perfusion imaging for the diagnosis of pulmonary thromboembolism **(DK)**

Differential pulmonary lung function studies **(BK)**

Mucociliary clearance studies **(BK)**

Lung permeability studies **(BK)**

***GI tract*** **(BK)**

Salivary gland imaging

Oesophageal and intestinal transit studies

Meckel's diverticulum studies

Radiolabelled red blood cell studies (GI bleeding, liver haemangioma)

Liver RES scintigraphy (both dynamic and static studies)

Hepatobiliary studies

Inflammatory bowel disease

Understanding of the role of breath tests in the investigation of malabsorption

***Urogenital tract*** **(BK)**

Dynamic renography (differential and absolute function, dilated outflow tract, obstruction, renovascular hypertension)

DMSA studies (pyelonephritis, ectopic kidneys, differential function)

GFR and ERPF measurement (blood and urine samples, computer-based techniques)

Indirect and direct cystourethrography

Understanding of related general issues, e.g. concurrent medication and preparation of the patient for the study, including the use of pharmaceutical interventions (e.g. ACEI, AT receptor blockade, furosemide)

***Endocrine*** **(BK)**

Benign thyroid disease, thyrotoxicosis, thyroid nodules

Interaction of agents such as amiodarone, iodine and drugs used to treat thyroid disease with radiopharmaceuticals

Malignant thyroid disease (understanding of the role of radio-iodine)

Parathyroid disease (understanding of the role of MIBI and SPECT/CT)

Neuroendocrine tumours (understanding of the role of SPECT/CT, if required in addition to planar imaging, and of PET/CT)

Adrenal imaging

***Lymphatic system***

Lymphoedema studies **(BK)**

Sentinel lymph node identification (breast, melanoma, head and neck, urogenital tumours) **(DK)**

Lymphatic leaks **(BK)**

***Infection/inflammation (DK)***

Processes involved in infection/inflammation

Understanding of the clinical role of different tracers in:

Bone scintigraphy (multiphase imaging)

HMPAO WBCs

Indium WBCs

Gallium citrate

Antigranulocyte antibodies (intact and fragments) FDG

Understanding the role of SPECT/CT, if required in addition to planar imaging, and of PET/CT

***Oncology (DK)***

Understanding of tumour biology

Understanding of the clinical indications for the various tracers used:

Radiolabelled antibodies

Pentavalent DMSA

Peptides

MIBG

MIBI

FDG

Other PET tracers

Understanding of the role of PET/CT and of SPECT/CT, if required in addition to planar imaging, in tumour imaging

***Paediatrics***

Normal anatomy and physiology to include knowledge of growth and maturation **(BK)**

Specific uses of nuclear medicine in paediatrics **(BK)**

Special precautions/protocols/legislation used in paediatrics (sedation, immobilisation, scintigraphic techniques) and adjustment of administered activities **(DK)**

Understanding specific issues in children such as immobilisation and radioprotection **(DK)**

**C. Topics specific to PET/CT**

Understanding of general issues related to preparation of the patient for the study **(DK)**

***Oncology***

Tumour metabolism **(DK)**

The specific patterns of spread for the various tumours **(DK)**

Normal distribution of F-18-FDG, C-11 and F-18 choline, C-11 methionine, NaF-18 and Ga-68 peptides **(DK)** The patterns of non-malignant disease including thyroid nodules, muscle uptake, brown fat, ovarian uptake, benign tumours, infection, inflammation and other potential pitfalls **(CS)**

Specific uses of FDG PET **(CS):**

Lung cancer

Lymphoma

Bowel cancer

Malignant melanoma

Oesophagus/stomach cancer

Head and neck cancer

Cervical/ovarian cancer

Cancer of unknown primary

Sarcoma

Thyroid cancer

Breast cancer

Pancreatic cancer

Renal cancer

Testicular tumours

GIST

Non-malignant uptake associated with the above tumours: **(CS)**

Infection

Sarcoid

Inflammation (reflux oesophagitis, rheumatoid joint disease, seronegative spondylarthropathies, tonsillitis, surgical wounds, post-radiotherapy)

Thymic rebound

Recurrent laryngeal nerve palsy

Bone marrow uptake after chemotherapy

Malignancies in which FDG-PET has a limited role (low-grade malignancies) **(DK)**

Specific uses of C-11 and F-18-choline in prostate cancer **(DK)**

Specific uses of C-11 methionine, F-18 FET and FDG in brain tumours **(BK)**

Specific uses of Ga-68 peptides and F-18 FDOPA in neuroendocrine tumours **(BK)**

The role of PET in detection, staging, follow-up and assessment of recurrence, and the relation of PET/CT to other imaging investigations **(CS)**

Specific protocols used in the different types of cancer (time of image acquisition, anatomical field to be examined, arms up or down, medication etc.) **(CS)**

Response assessment in the various tumours and expected patterns according to the applied treatment (surgery, radiotherapy, chemotherapy, targeted therapy) **(CS)**

***Benign indications (CS)***

Specific uses of FDG PET in infection and inflammation (vascular, musculoskeletal, fever of unknown origin, granulomatous diseases)

**PART III**

**Radiology Curriculum for those whose Training Background is in Nuclear Medicine**

**Preamble to Part III**

In 2011 the “Multimodality Imaging Training Curriculum – General Recommendations” were published in the *EJNMMI* (Eur J Nucl Med Mol Imaging DOI 10.1007/s00259-011-1804-2) and in *Insights into Imaging* (Insights Imaging 2(2): 99-101, DOI: 10.1007/s13244-011-0067-6). Both the European Association of Nuclear Medicine and the European Society of Radiology committed themselves to writing more detailed recommendations regarding skills and knowledge requirements for nuclear medicine physicians in radiology and radiologists in nuclear medicine. Parts II & III of the Multimodality Imaging Training Curriculum were endorsed by the ESR Executive Council in June 2011, as well as the EANM Executive Committee, the EANM Advisory Council and on February 26, 2012 by the extraordinary EANM Delegates Assembly.

The Multimodality Imaging Training Curriculum is a joint European initiative between the EANM and the ESR to define the scope of training for medical specialists in hybrid imaging. The curriculum is a framework outline that may be used by member societies where appropriate to form the basis for discussion on the optimisation of further training of medical specialists.

This curriculum cannot be used to legally justify changes to the organisation, overall specialist training or reimbursement regulations in any member state.

**Radiology Curriculum for those whose Training Background is in Nuclear Medicine**

The following outlines a check list of radiology training for nuclear medicine physicians to ensure the appropriate level of knowledge is obtained for performing and reading hybrid imaging such as PET/CT and SPECT/CT within a two years training period. This checklist differentiates three levels of knowledge: basic knowledge (BK), detailed knowledge (DK), and core skills (significant practical experience required, CS).

**A. Required general training in radiology**

- Principles of imaging technology as applied to diagnostic radiology (radiation physics) **(BK)**
- Radiobiology **(BK)**
- The physical basis of image formation including conventional x-ray, computed tomography, magnetic resonance imaging **(DK)**
- Radiographic quality control **(DK)**
- Radiation protection **(DK)**
- Anatomy, normal variants, pathological features, physiology, biochemistry and techniques related to radiological procedures **(BK)**
- Cell biology, DNA, RNA, and cell activity **(BK)**
- Clinical indications for the administration of intravenous and oral contrast media **(BK)**
- Basic principles of image acquisition and post-processing to include MPR, MIP and volume rendering principles; understanding of concepts of data acquisition in CT technology and volumetric CT data acquisition; knowledge of detector composition and physics, CT numbers and evaluation of image quality including field size and spatial and contrast resolution; familiarity with major CT artefacts including motion, streak, beam hardening and ring artefacts **(DK)**
- Basic understanding of computer science, image archiving and image communication and teleradiology **(BK)**
- Clinical Application and Study Protocols to include: an understanding of patient preparation for PET/CT examinations, venous access and principles of the use of beta-blocking when appropriate **(DK)**
- Intravascular contrast media to include pharmacology, osmolar types, potential vascular toxicity, soft tissue toxicity, cardiovascular toxicity, neurotoxicity, nephrotoxicity and effects on thyroid function tests. A knowledge of idiosyncratic reactions to include incidence, distinction of minor, intermediate and major reactions, mechanisms of idiosyncratic reactions and principles of prophylaxis for adverse reactions to contrast media

**B. Specific studies by organ**

***Musculoskeletal systems***

Musculoskeletal anatomy, normal variants which may mimic disease and radiological appearances of common congenital dysplasias **(BK)**

Radiologic appearances of common arthropathies **(BK)**

Degenerative disorders and their clinical relevance **(DK)**

Manifestations of musculoskeletal infection, inflammation and metabolic diseases including osteoporosis and bone densitometry **(DK)**

Recognition, staging and surveillance of bone and soft- tissue tumours and haemotological disorders **(DK)**

Reporting CT and MR of common musculoskeletal disorders **(CS)**

Managing and reporting CT and MR of musculoskeletal trauma **(BK)**

***Thorax***

Features on CT and differential diagnosis of atelectasis and diffuse lung disease **(DK)**

Mediastinal and hilar lymph nodes: normal appearance, normal dimensions, causes of calcification, radiological features of adenopathy on CT to include size criteria, features of reactivity and lymphatic drainage pathways, knowledge of American Thoracic Society lymph node location nomenclature **(CS)**

Features of pneumothorax including: evidence of tension, pneumomediastinum etc **(BK)**

Solitary and multiple pulmonary nodules: definition, recognition of micronodular, nodular, macronodular and cavitating nodular lung, disease with differential diagnosis, understand role of radiology in follow-up lung nodule, features of benignity within pulmonary nodules **(CS)**

Protocols for evaluation for single and multiple pulmonary nodules **(DK)**

Malignant neoplasms of the lung, including knowledge of: histological types, TNM, features of recurrent tumour post treatment or surgery **(CS)**

The manifestations and role of imaging in thoracic lymphoma **(CS)**

Interpretation of high resolution thoracic CT specifically with reference to diffuse lung disease and exclusion of opportunistic lung disease **(CS)**

Recognition of features of pulmonary embolism on CT/PA **(CS)**

***Gastrointestinal***

Knowledge of radiologic appearances in viscous perforation, inflammation, infection, obstruction, ischaemia and infarction on plain radiographs, contrast studies, and CT **(BK)**

Role of ultrasound and relevant ultrasound features in biliary tract and parenchymal solid organ disease **(BK)**

Imaging features and differential diagnosis of primary and secondary tumours of the solid organs, oesophagus, stomach, small bowel, colon and rectum **(DK)**

Imaging features of the stage and extent of tumours including features which indicate unresectability and knowledge of the role of endoscopy and endoscopic US **(DK)**

Radiological manifestations of inflammatory bowel diseases, malabsorption syndrome and infection **(BK)**

Recognition of motility disorders, hernias and diverticula **(BK)**

Radiological manifestation of vascular lesions including varices, ischaemia, infarction, haemorrhage and vascular malformations **(BK)**

An understanding of the role of CT colonoscopy **(BK)**

Understanding of the applications of angiography, vascular interventional techniques, stenting and portosystemic decompression procedures **(BK)**

Tumours, infectious and degenerative lesions of the liver **(DK)**

Managing and reporting CT of the abdomen **(CS)**

Experience of the manifestations of abdominal disease on MRI **(CS)**

***Urogenital***

Understanding of renal function, the diagnosis of renal parenchymal diseases including infection and renovascular disease and management of renal failure **(BK)**

Imaging features and appropriate investigation of calculus disease **(BK)**

Investigation and features of urinary tract obstruction and reflux **(BK)**

Imaging features and differential diagnosis of tumours of the kidney and urinary tract **(DK)**

Imaging features and differential diagnosis of the diseases of the retroperitoneum, prostate and testis **(DK)**

Managing and reporting computed tomography and MR imaging of the retroperitoneum, urinary tract and pelvis **(CS)**

**Interventional radiology and vascular system**

A knowledge of the role of interventional techniques specifically with reference to procedures such as tumour biopsy, embolisation, biliary drainage, pleural effusion aspiration etc. **(BK)**

A knowledge of radiological methods related to the vascular and lymphatic system (**BK**)

***Paediatric***

Normal paediatric anatomy and normal variants with particular focus on normal maturation and growth **(BK)**

Disease entities specific to the paediatric age group and their clinical and radiological manifestations using all potential imaging investigations **(BK)**

The value and indications for CT and MR in children **(DK)**

Disorders and imaging features of the neonate **(BK)**

Managing and reporting CT and MR examinations **(CS)**

Detailed knowledge of ALARA principle and its application in a paediatric population **(BK)**

Particular benefits of ultrasound in evaluation of disease in the paediatric population **(BK)**

Knowledge of benign disease which may mimic malignancy **(DK)**

***Head and neck***

Knowledge of normal anatomy and variants of facial bones and paranasal sinus anatomy **(BK)**

Features of benign disease of mandible and maxilla which mimic malignancy disease **(DK)**

Diagnosis of faciomaxillary trauma and tumours **(DK)**

Radiology of disorders of thyroid, parathyroid glands and complementary evaluation with nuclear medicine techniques **(BK)**

Role of intravenous contrast radiology in evaluation of head and neck pathology **(CS)**

Imaging features of trauma, inflammation, infection and tumours of the paranasal sinuses, oral cavity, larynx and pharynx **(DK)**

Managing and reporting CT and MR of neck, ear, nose, throat and skull base disorders **(CS)**

***Gynaecology and obstetrics***

Imaging features of disorders of the ovaries, uterus and vagina as demonstrated on CT and MR **(BK)**

Awareness of specific benefits of ultrasound in evaluation and interventional technique for gynaecologic malignancy **(BK)**

Awareness of the applications of angiography and vascular interventional techniques **(BK)**

***Oncology***

Tumour classification and staging nomenclature **(DK)**

Application of all imaging and interventional techniques in staging and monitoring the response of tumours to therapy **(DK)**

Knowledge of expected radiologic change in response to therapy **(DK)**

Radiological manifestations of complications in tumour management **(DK)**

Performing and reporting CT, and MR to assess tumours and response to therapy **(CS)**

Principles of communication as described in Part I of this document (EJNMMI 38: 976–978; DOI 10.1007/s00259-011-1804-2)

